# Prolonged intensive care therapy in nonagenarians admitted to the intensive care unit—clinical characteristics, risk factors and outcomes

**DOI:** 10.3389/fmed.2025.1728917

**Published:** 2026-01-12

**Authors:** Markus Haar, Jakob Müller, Rikus Daniels, Pauline Theile, Stefan Kluge, Kevin Roedl

**Affiliations:** 1 Department of Intensive Care Medicine, University Medical Centre Hamburg-Eppendorf, Hamburg, Germany; 2 Department of Anaesthesiology, Tabea Hospital, Hamburg, Germany

**Keywords:** critical illness, critically ill, ICU, nonagenarians, prolonged ICU stay, very elderly

## Abstract

**Introduction:**

Intensive care unit (ICU) admissions of very elderly patients (≥90 years) have increased in recent years. To date, it remains unclear if prolonged ICU treatment (≥7 days) is justified regarding outcome. Yet, factors associated with prolonged ICU stay remain unknown in the very elderly critically ill population.

**Materials and methods:**

This retrospective study analysed all adult patients aged ≥90 years consecutively admitted to the ICU at a tertiary care centre in Hamburg, Germany, (01/01/2008–03/31/2019). Multivariable regression and Kaplan–Meier estimates were employed to assess the independent predictors of a prolonged ICU stay.

**Results:**

Of 1,091 very elderly patients admitted to the ICU, 10% (*n* = 110) experienced a prolonged ICU stay (≥7 days). Demographic characteristics, including age, gender, and body mass index (BMI)—were similar across groups. Patients with extended stays had higher admission SAPS II and SOFA scores (47 vs. 35 and 5 vs. 2 points, respectively, *p* < 0.001 for both). The requirement for mechanical ventilation (MV) and renal replacement therapy (RRT) was higher in the prolonged stay group (78 and 12%, respectively) compared to the shorter-stay group (30 and 2%, respectively, *p* < 0.001 for both). Multivariate regression analysis identified MV [OR 3.873, 95% CI (2.026–7.406); *p* < 0.001], vasopressors [OR 2.921, 95% CI (1.466–5.821); *p* = 0.002], RRT [OR 4.299, 95% CI (1.513–12.212); *p* = 0.006] and SAPS II [OR 1.023, 95% CI (1.004–1.043); *p* = 0.020] as independent intra ICU markers associated with a prolonged stay. ICU and hospital mortality rates were higher in the prolonged stay group (32 and 50%, respectively) compared to those with shorter stays (17 and 28%, respectively, *p* < 0.001 for all).

**Conclusion:**

Approximately 10% of critically ill patients aged 90 years and above experienced prolonged ICU stays which was significantly associated with increased mortality. Prolonged ICU therapy has little long-term benefit and high mortality. Therefore, providing prolonged ICU treatment must be carefully considered incorporating individual frailty, premorbid function, and goals of care.

## Background

The global population is currently experiencing a decrease in fertility rates alongside increased life expectancy, leading to a rapid aging demographic. A significant aspect of this shift is the growth in the population of very elderly individuals, with projections indicating that by 2030, the number of nonagenarians will surpass 30 million (1). Due to this demographic development and advancements in providing medical care, the proportion of very elderly patients admitted to the intensive care unit (ICU) is expected to rise substantially. Presently, approximately 15% of critically ill patients in ICU present at an advanced age of 80 years and above; 1% was observed to be beyond 90 years of age (2, 3). While studies have reported acceptable outcomes among this age group, debates on limitation of therapy and futility of care in this age group remain subject of considerable debate (3, 4). Nonetheless, age alone should be a determining factor in withholding critical care (3).

The median length of ICU stay in nonagenarians ranges from 1.4 to 6.1 days, depending on the patient groups studied, however, a majority can be discharged within 7 days or less (3, 5, 6). In contrast, a prolonged ICU stay is generally associated with increased mortality and unfavourable functional outcomes (7–9). With continuous advancements in medical treatments, it is anticipated that an increasing number of patients will experience extended ICU stays, including those at advanced age (10). This will pose challenges in decision-making concerning treatment intensity and futility of care, particularly in cases with unanticipated outcomes. Among the general ICU population, age was identified as relevant risk factor in patients with very prolonged ICU stays (11–13). Notably, prolonged mechanical ventilation (MV) as well as length of ICU stay (LOS) are associated with impaired long-term survival and unfavourable quality of life, especially in the very elderly (7–9, 14–16). Additionally, functional outcomes post-prolonged critical illness tend to be poorer and often persistent, necessitating careful consideration in the management of these cases (15). Despite these concerns, there remains a notable scarcity of studies on the characteristics and outcomes of nonagenarians, specifically in those with an extended ICU stay.

Therefore, the primary objective of this study was to investigate the incidence of prolonged ICU stay (≥7 days). Further clinical characteristics, risk factors, and outcomes to identify patients at risk for a prolonged intensive care therapy will be investigated.

## Patients and methods

### Study design, setting and ethics

Data of all adult patients ≥90 years consecutively admitted to the Department of Intensive Care Medicine at the University Medical Centre Hamburg-Eppendorf (Germany) between January 2008 and March 2019 were analysed. The department constitutes of 12 intensive care units (ICU) and cares for all critically ill adult patients of the hospital with a total capacity of 140 beds.

### Inclusion and exclusion criteria

All adult patients (≥90 years) admitted to the ICU were included in the study. All patients <90 years of age, or patients with incomplete clinical data were excluded.

### Data collection

Data were documented prospectively by trained staff. Second, we show results of a large tertiary care centre experienced in the management of critically ill patients. Data was then collected through electronic patient data management system (PDMS, Integrated Care Manager^®^ (ICM)—Draeger Medical, Luebeck, Germany). The extracted data included age, sex, comorbidities, admission diagnosis, length of ICU- and hospital-stay, outcome, treatment modalities and organ support (mechanical ventilation, vasopressor, renal replacement therapy, blood transfusions, antibiotics, antivirals, etc.) and laboratory parameters. Laboratory assessment was performed on daily basis within clinical routine.

### Study definitions and patient management

Prolonged ICU stay was defined as continuous presence in the ICU for a period of 7 days or more. To evaluate the severity of illness upon admission, the Sequential Organ Failure Assessment (SOFA) (17) and Simplified Acute Physiology Score II (SAPS II) (18) were utilised. The Charlson comorbidity index (CCI) (19) was calculated for each patients to assess their comorbidity burden.

### Study definitions and patient management

We defined incidence of prolonged ICU stay (≥7 days) as primary outcome. Furthermore, we defined ICU mortality, hospital mortality, 1-year mortality, hospital length of stay as secondary outcomes.

### Statistical analysis

Data are presented as absolute numbers and relative frequency or median with interquartile range (IQR) as appropriate. Categorical variables were compared using either chi-square-analysis or Fisher’s exact test. For continuous variables, Mann–Whitney-*U* test was employed. Exposures were assessed either at ICU admission or at any time during the ICU stay, as specified for each variable. Some exposures are time-dependent, and competing risks were considered when interpreting associations. Patient factors associated with the occurrence of prolonged ICU stay and mortality were assessed. Therefore, multivariable logistic regression analysis using clinically available variables was performed employing a stepwise backward elimination approach to derive the model; variables that exhibited a change in estimates >10% and with maintained statistical significance were included in the model. Statistical analysis was conducted using IBM SPSS Statistics Version 24.0 (IBM Corp., Armonk, NY). Throughout the analysis, a *p*-value <0.05 was deemed statistically significant. The study was prepared in accordance with the STROBE (STrengthening the Reporting of OBservational studies in Epidemiology) recommendations (20).

## Results

### Overview and primary outcome

**Figure 1:**
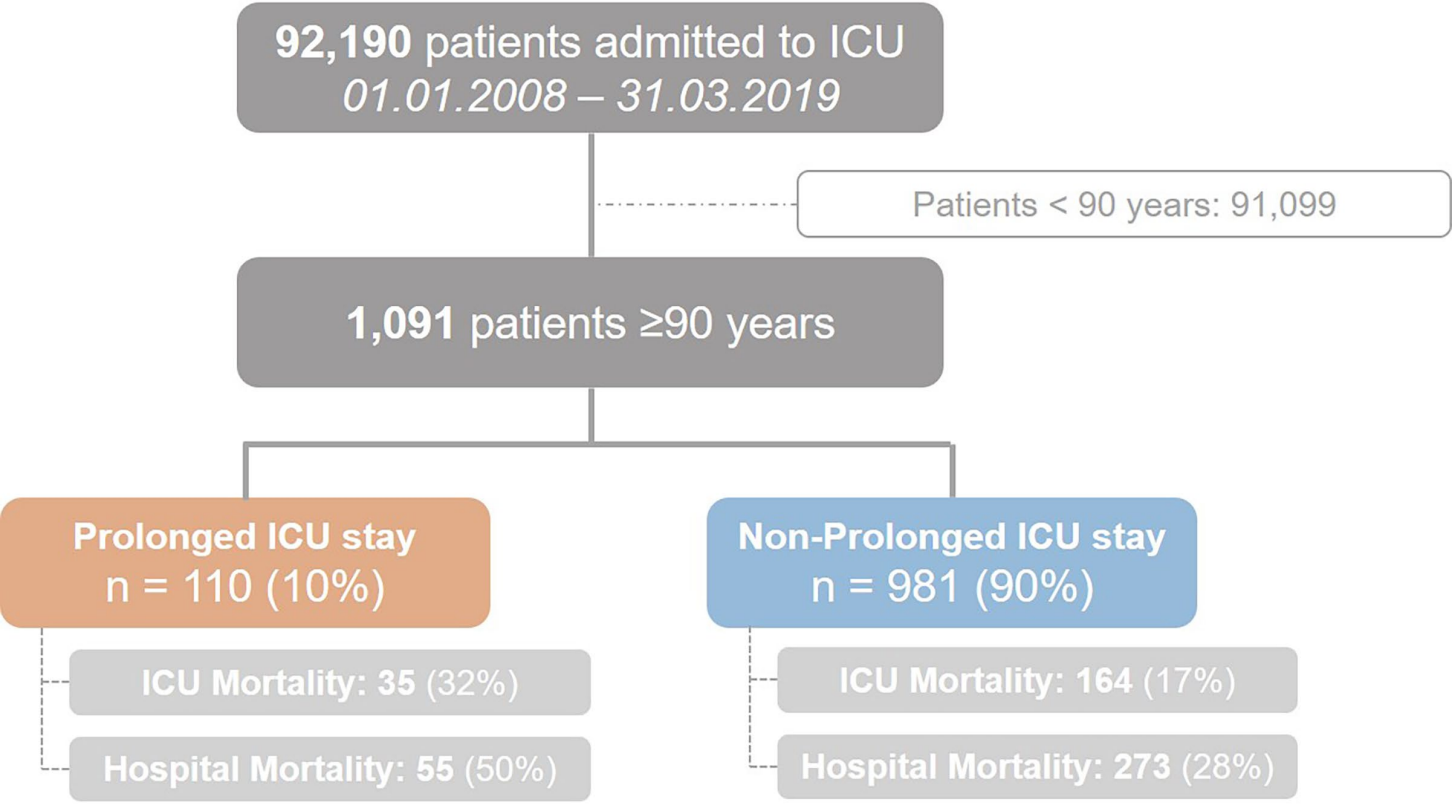
Flow chart of the study.

During the study period from January 1st, 2008 to March 31st, 2019 a total number of 92,190 patients were treated at the department of intensive care medicine. After the exclusion of 17 cases due to incomplete data 1,091 patients ≥90 years were identified and included in this study (see flow chart—Figure 1). In this study the primary outcome is incidence of a prolonged ICU stay. Of 1,091 very elderly patients admitted to the ICU, 10% (*n* = 110) experienced a prolonged ICU stay (≥7 days).

### Study population

The median age of the patients was 92.3 years (IQR: 91.0–94.2) and 32% (*n* = 350) were male. The median body mass index was 23.4 kg/m^2^ (IQR: 21.0–26.0). Patients were admitted to the ICU due to medical reasons (34%, *n* = 373), elective surgery (37%, *n* = 402), and emergency surgery (28%, *n* = 309) surgery. The severity of disease, as represented by the SAPS II and SOFA score, had a median of 36 points (IQR: 28–47) and 2 points (IQR: 1–5) on admission, respectively. The burden of disease as assessed by the CCI was 1 (IQR: 0–2) was observed in our cohort. Arterial hypertension (70%, *n* = 762), chronic kidney disease (23%, *n* = 249) and congestive heart failure (22%, *n* = 241) were the most frequent comorbidities observed. On the day of admission, 22% (*n* = 238) received invasive mechanical ventilation (MV) and non-invasive mechanical ventilation (NIV) or high-flow nasal cannula (HFNC) in 7% (*n* = 77). Additionally, vasopressor therapy was necessary in 33% (*n* = 363) of patients. The median duration of ICU and hospital stay was 1.6 (IQR: 0.9–3.5) and 11.0 (7.0–16.6) days, respectively. The ICU mortality was 18% (*n* = 199) and the hospital mortality was 30% (*n* = 328) (see Table 1).

**Table 1:** Baseline characteristics of the study population.

Variables	All (*n* = 1,091)
Age (years)	92.3 (91.0–94.2)
Males	350 (32)
Weight (kg)	65 (55–74)
Height (cm)	165 (160–170)
BMI (kg/m^2^)	23.4 (21.0–26.0)
Primary admission	
Medical	373 (34)
Surgical—planned	402 (37)
Surgical—emergency	309 (28)
Disease severity	
SAPS II—admission (pts.)	36 (28–47)
SOFA—admission (pts.)	2 (1–5)
Comorbidities	
Charlson comorb. index, pts.	1 (0–2)
Arterial hypertension (*n*, %)	762 (70)
Chronic kidney disease (*n*, %)	249 (23)
Coronary heart disease (*n*, %)	169 (16)
Congestive heart failure (*n*, %)	241 (22)
Diabetes mellitus (*n*, %)	148 (14)
Chronic lung disease (*n*, %)	88 (8)
Vital functions—admission	
Body temperature (C°)	36.2 (35.5–36.8)
Heart rate (beats/min)	80 (65–97)
Mean arterial pressure (mmHg)	86 (72–102)
Vasopressor use	363 (33)
Respiratory support—admission	
Invasive mechanical ventilation	238 (22)
Non-invasive ventilation/HFNC	77 (7)
Outcome	
Duration ICU stay (days)	1.6 (0.9–3.5)
Duration hospital stay (days)	11.0 (7.0–16.6)
Died in ICU	199 (18)
Died in hospital	328 (30)

Data are expressed as *n* (%) or median (interquartile range).

kg, kilogram; m, meter; ICU, intensive care unit; BMI, body mass index; pts., points; HFNC, high-flow nasal cannula.

### Occurrence and clinical characteristics of patients with and without prolonged intensive care unit stay

Detailed characteristics are demonstrated in Table 2. Demographic characteristics including age, gender and BMI were comparable in patients with and without prolonged ICU stay. Patients with prolonged ICU stay were admitted to the ICU due medical reasons (49% vs. 33%, *p* = 0.001), elective surgical (25% vs. 38%, *p* = 0.009) and emergency surgical (24% vs. 29%, *p* = 0.250) as compared to patients without prolonged ICU stay, respectively. The median CCI did not differ between both groups [1 (IQR: 0–3) vs. 1 (IQR: 1–2), *p* = 0.933]. Detailed comorbidities are presented in Supplementary Table 1. At admission SAPS II [47 (IQR: 37–65) vs. 35 (IQR: 27–45), *p* < 0.001] and SOFA [5 (IQR: 3–9) vs. 2 (IQR: 1–5), *p* < 0.001] were higher in patients with a prolonged stay; as they were 24 h after admission [SOFA: 5 (IQR: 3–10) vs. 2 (IQR: 1–4)].

**Table 2:** ICU characteristics of patients with and without prolonged ICU-stay.

Variables	Prolonged stay (≥7 days) (*n* = 110)	Non-prolonged stay (<7 days) (*n* = 981)	*p*-value
Age (years)	92.2 (90.8–93.8)	92.3 (91.0–94.2)	0.389
Males	43 (39)	307 (31)	0.097
BMI (kg/m^2^)	23.4 (21.5–26.2)	23.4 (20.8–26.0)	0.624
Primary admission			
Medical	54 (49)	319 (33)	0.001
Surgical—planned	28 (25)	374 (38)	0.009
Surgical—emergency	26 (24)	283 (29)	0.25
Disease severity			
Charlson comorb. index, pts.	1 (0–3)	1 (1–2)	0.933
SAPS II—admission (pts.)	47 (37–56)	35 (27–45)	<0.001
SOFA—admission (pts.)	5 (3–9)	2 (1–5)	<0.001
SOFA—24 h (pts.)	5 (3–10)	2 (1–4)	<0.001
Respiratory support			
Invasive MV	86 (78)	296 (30)	<0.001
Duration of MV (days)	2.6 (0.7–6.2)	0.4 (0.2–1.0)	<0.001
Procedures/Therapies			
Vasopressors	92 (84)	366 (37)	<0.001
Renal replacement therapy	13 (12)	17 (2)	<0.001
Parenteral Nutrition	21 (19)	57 (6)	<0.001
Cardiopulmonary resuscitation	18 (16)	64 (7)	<0.001
Hypoxic liver injury	10 (9)	25 (3)	<0.001
Jaundice >3 mg/dL	4 (4)	9 (1)	0.013
Outcome			
Duration ICU stay (days)	10.1 (8.1–12.8)	1.3 (0.9–2.6)	<0.001
Duration hospital stay (days)	16.1 (11.7–20.4)	10.2 (6.3–16.0)	<0.001
Died in ICU	35 (32)	164 (17)	<0.001
Died in hospital	55 (50)	273 (28)	<0.001

Data are expressed as *n* (%) or median (interquartile range).

ICU, intensive care unit; BMI, body mass index; kg, kilogram; m, meter; pts., points; mv, mechanical ventilation; LDH, lactate-dehydrogenase; CRP, C-reactive protein.

The need for invasive mechanical ventilation was higher [78% (*n* = 86) vs. 30% (*n* = 296), *p* < 0.001] and ventilation episodes were longer [2.6 days (IQR: 0.7–6.2) vs. 0.4 days (IQR: 0.2–10), *p* < 0.001] in the subgroup of patients with a prolonged ICU stay. 84% (*n* = 92) and 37% (*n* = 366) patients with and without prolonged ICU stay received vasopressor therapy, respectively (*p* < 0.001). Twelve percent (*n* = 13) in the prolonged ICU stay group underwent RRT, while this was necessary in only 2% (*n* = 17) of patients with shorter ICU stays (*p* < 0.001). Parenteral nutrition therapy, alone or together with enteral nutrition, was applied in 19% (*n* = 21) with prolonged ICU stay and in 6% (*n* = 57) without prolonged ICU stay (*p* < 0.001). The ICU stay was complicated due to hypoxic liver injury (9% vs. 3%, *p* < 0.001), jaundice (4% vs. 1%, *p* < 0.001) and cardiopulmonary resuscitation (16% vs. 7%, *p* < 0.001).

### Laboratory and blood gas analysis on admission

On admission, haemoglobin (10.4 vs. 10.3 g/dL, *p* = 0.975), and leukocyte (11.6 vs. 10.7 /nL, *p* = 0.057) levels were comparable, while LDH levels were found to be significantly higher in the prolonged stay group (292 vs. 251 U/L, *p* = 0.011); furthermore, both CRP [59 (IQR: 17–125) vs. 27 (IQR: 8–75) mg/L, *p* = 0.001] and serum creatinine [1.3 (0.8–2.0) vs. 1.1 (0.8–1.6) mg/dL, *p* = 0.005] were higher. Median thrombocyte and bilirubin levels were comparable and in normal ranges in both groups. Parameters obtained from arterial blood gas analysis pH, bicarbonate, oxygen and carbon dioxide partial pressures were similar in both groups. In contrast, median serum lactate levels differed slightly [1.3 (IQR: 0.9–2.3) vs. 1.1 (0.8–1.7) mmol/L, *p* = 0.002] at admission, while this difference was more pronounced comparing their median peak serum level [2.4 (1.8–3.9) vs. 1.7 (1.1–2.6) mmol/L, *p* < 0.001]. For detailes please see Supplementary Table 2.

### Factors during the ICU stay associated with a prolonged stay

Multivariate regression analysis identified mechanical ventilation [OR 3.873, 95% CI (2.026–7.406); *p* < 0.001], requirement of vasopressors [OR 2.921, 95% CI (1.466–5.821); *p* = 0.002], RRT [OR 4.299, 95% CI (1.513–12.212); *p* = 0.006], and SAPS II [OR 1.023, 95% CI (1.004–1.043); *p* = 0.020] were identified as associated intra ICU markers with a prolonged stay (see Table 3).

**Table 3:** Multivariable logistic regression for factors associated with occurrence of prolonged ICU stay.

Logistic regression	Covariables	OR (95% CI)	*p*-value
Final model	SAPS II	1.023 (1.004–1.043)	0.020
	Mechanical ventilation—during ICU (yes vs. no)	3.873 (2.026–7.406)	<0.001
	Vasopressors—during ICU (yes vs. no)	2.921 (1.466–5.821)	0.002
	Renal replacement therapy—during ICU (yes vs. no)	4.299 (1.513–12.212)	0.006

Multivariable logistic regression for factors associated prolonged ICU stay (variables included in the initial model: gender, Simplified Acute Physiology Score (SAPS) II at admission, Charlson comorbidity index, mechanical ventilation during ICU, vasopressors during ICU, Sepsis-related Organ Failure Assessment (SOFA) score, body-mass-index, renal replacement therapy during ICU). OR, odds ratio; CI, confidence interval; SAPS II, Simplified Acute Physiology Score II; ICU, intensive care unit.

### Outcomes and secondary outcomes of patients with and without prolonged intensive care unit stay

The median length of stay in the ICU was significantly longer in the prolonged ICU stay group, recorded at 10.1 days (IQR: 8.1–12.8), compared to 1.3 days (IQR: 0.9–2.6) in the shorter stay group. Similarly, the median hospital stay was also notably longer, being 16.1 days (IQR: 11.7–20.4) in the prolonged stay group vs. 10.2 days (IQR: 6.3–16.0) in the other group (*p* < 0.001 for both).

**Figure 2:**
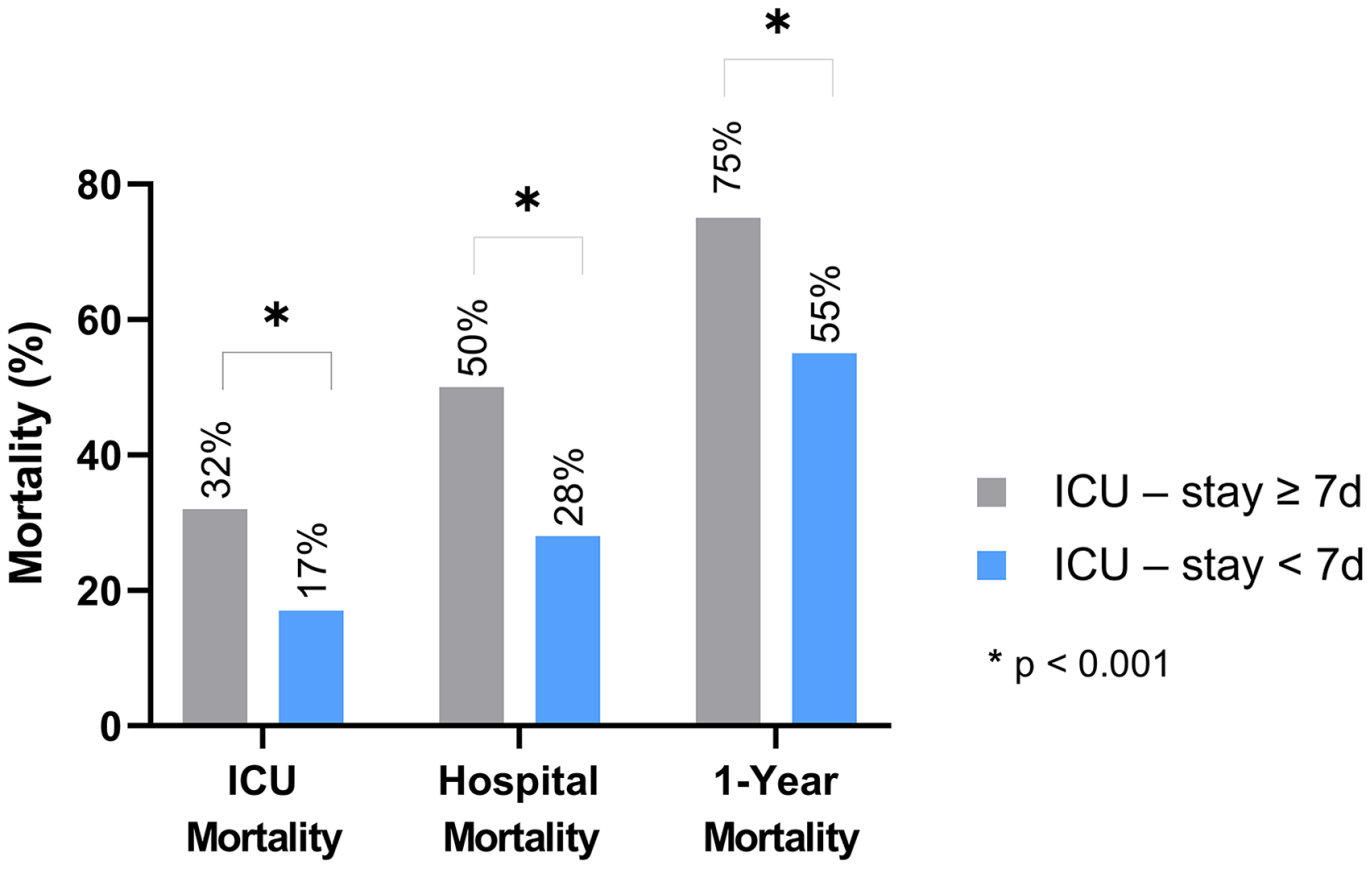
Survival stratified according prolonged and non-prolonged ICU-stay.

In the cohort of patients with prolonged ICU stays, the observed ICU, hospital, and 1-year mortality rates were notably higher, at 32, 50, and 75%, respectively; compared to 17, 28, 55% and in patients without prolonged ICU stays (*p* < 0.001 each). Furthermore, the one-year long-term mortality rate was significantly greater in patients with prolonged ICU stays, standing at 75% (*n* = 83), as opposed to 55% (*n* = 541) in those with non-prolonged stays (refer to Figure 2).

## Discussion

In this study of patients ≥90 years admitted to the ICU, we found that 10% of the patients required a prolonged ICU stay (≥7 days) which was associated with a significantly higher short- and long-term mortality. To our knowledge, this is the first study investigating occurrence, outcome and factors associated with presence of prolonged ICU stay among very elderly critically ill patients.

Across all ages, the average ICU length of stay usually ranges between 1.4 to 6.1 days, with the majority of patients requiring a stay below 7 days (3, 5, 6, 21). Additionally, for elderly patients not requiring critical care, prolonged hospital stays have been reported to be associated with an increased rate of complications and unfavourable outcome, including hospital re-admission, mortality, as well as deterioration of functional status (22). In their paper on the syndrome of the “chronically critically ill,” Girard and Raffin (23) described a patient group surviving an initial episode of critical illness, however, failing to recover beyond the point of becoming independent of critical care (“neither dying nor recovering”). Respiratory failure and prolonged dependence on invasive ventilation constitute the hallmarks of chronic critical illness. It is estimated that between five to 10% of patients who undergo invasive ventilation initially will eventually become part of this chronically critically ill group (11, 24). It appears reasonable to expected that this proportion will be significantly higher the group of nonagenarians, especially in those with a prolonged ICU stay.

The population of patients with a prolonged stay in the ICU is consuming a high number of ICU resources (25–29). This issue takes on particular significance in the context of limited bed capacity, which is often exacerbated by constraints in nursing staff availability. Furthermore, while most published studies report to be favourable cost-efficiency ratios (less than $50,000 per quality-adjusted life year), a review by Wilcox et al. (30) found that 17% (*n* = 20) of studies indicated cost-efficiency-ratios surpassing $100,000 per quality adjusted life year.

Recent trends in population aging, along with advancements in critical care management, have been identified as key factors contributing to the increasing number of patients experiencing prolonged ICU stays (1, 10, 11). However, due to the large heterogeneity within this patient group, a universally accepted definition of “prolonged ICU stay” has yet to be established. In the distinct cohort of nonagenarians investigated in this study, an ICU stay beyond 7 days was observed in 10%. In those, a clear association with an underlying medical condition could be established, affecting approximately half of them.

In this study, the overall severity of illness was low. Nonetheless, patients admitted with a higher SOFA and SAPS II scores were at increased risk for prolonged ICU stays. Notably, during their extended time in the ICU, more than 80% of patients required vasopressor support. It is important to note, though, that this high proportion may be influenced by the inclusion of patients suffering cardiac arrest, with potential high rates of post-cardiac arrest shock or heart failure (31, 32).

Interestingly, we observed a high rate of hypoxic liver injury and cholestasis in patients with a prolonged ICU stay. This might be attributed to the common co-occurrence of hypoxic liver injury in severe illness and might be observed in settings of cardiac, circulatory, or respiratory failure (33, 34).

Acute kidney injury frequently complicates ICU stay, often mirroring severity of illness (35). Indeed, 10% of patients in the prolonged stay group required renal replacement therapy. The occurrence of organ failure was notably influential in the development of prolonged ICU stays in this cohort.

Due to the growing complexity of medical interventions, even in advanced age (such as trans-femoral aortic valve implantation, TAVI), along with the management of ensuing complications, as well as advancements in intensive care medicine, it is anticipated that a greater number of patients will experience prolonged lengths of stay in the ICU (10). This is accompanied with challenges in decision making on the treatment intensity and futility of care. Physical function and cognitive status following (prolonged) critical illness are frequently poorer and more enduring than initially anticipated: Studies reveal that less than 12% of chronically critically ill patients are alive and independent a year after acute illness. This situation places heavy burdens on families, leading to high rates of depression and caregiver burnout. Notably, the decline in caregivers’ physical health and the increased stress are prevalent not just in home care settings but are often at least as severe for families of institutionalized patients (11, 15).

Recent research underscores the significant impact of frailty on mortality among critically ill, very elderly patients (36, 37). Consequently, frailty should be a critical consideration in ICU treatment discussions. Although frailty was not directly assessed in our cohort, we noted that 61% of patients lived independently prior to their ICU admission, while the remainder resided in nursing homes or assisted living facilities, suggesting they might be more vulnerable and presumably frailer. Contrary to expectations, a recent study found the most significant impact of frailty on mortality risk in middle-aged patients, rather than in the very elderly. Consequently, frailty should not be the sole determinant in excluding very elderly patients from critical care therapy, highlighting the need for a more nuanced approach in decision-making (38).

Although, we observed an increased ICU- and 1-year mortality in patients with a prolonged ICU stay, survival rates were acceptable considering the advanced patient age. However, the clinical situation and potential deteriorations should be perceived early and treatment intensity should be adapted according patients and family wishes to optimise compassionate care while avoiding futile life-sustaining interventions.

This study has several limitations: First, we show the results of a retrospective single centre study alongside with its limitations. However, data were documented prospectively by trained staff. Second, we show results of a large tertiary care centre experienced in the management of critically ill patients. Therefore, results and conclusions may not be transferable to other settings. Third, in the logistic regression model we applied a stepwise selection method which can produce an unstable model and biased estimates, particularly in datasets with a limited number of events. However, we used a stepwise selection with the intend to explore associations in an observational dataset rather than to infer causality. Fourth, we acknowledge that some exposures are time-dependent and that competing risks may influence observed associations. The timing of exposure measurement—either at ICU admission or during the ICU stay—may limit causal interpretation and should be considered when applying these findings clinically. Fifth, residual confounding from unmeasured covariables is a matter of concern and cannot be entirely excluded.

## Conclusion

Our study demonstrated that prolonged ICU stays of nonagenarians are not uncommon and are associated with little long-term benefit, which, however, may be considered acceptable given the advanced age of the patients. Intra ICU markers associated with prolonged ICU stays included mechanical ventilation, vasopressor therapy, and renal replacement therapy. These findings suggest that prolonged ICU therapy for critically ill nonagenarians can be beneficial and should generally not be pre-emptively withheld. Due to limited physical reserves in this population, patients’ propensity to recover is low even when treated for a relatively short period of time in the ICU. Nevertheless, decisions regarding such therapy must be tailored, taking into account individual patient factors (e.g., individual frailty, premorbid function, and goals of care) for a more personalised approach, also integrating the expertise and realistic judgment of experienced medical professionals.

## Data Availability

The raw data supporting the conclusions of this article will be made available by the authors, without undue reservation.

## Supplementary material

Supplementary Table 1 Pre-existing comorbidities comparing prolonged stay vs. non-prolonged stay.

Supplementary Table 2 ICU Characteristics of patients with and without prolonged ICU-stay.
