# Supplementary material for: Prolonged intensive care therapy in nonagenarians admitted to the intensive care unit—clinical characteristics, risk factors and outcomes
Source: Front Med (Lausanne). 2026 Jan 12;12:1728917. doi: 10.3389/fmed.2025.1728917 (PMC12832867; doi:10.3389/fmed.2025.1728917)
Supplement: Supplementary file 1 [file Table_1.DOCX]

Supplementary Table 1 – Pre-existing comorbidities comparing prolonged stay vs. non-prolonged stay

| *Parameters* | *Prolonged stay (≥ 7 days) (n = 110)* | *Non-Prolonged stay (< 7 days) (n = 981)* | *p*-value* |
| --- | --- | --- | --- |
| AIDS *n (%)* | 0 (0) | 1 (1) | 0.899 |
| Cerebral arterial disease *n (%)* | 15 (14) | 152 (15) | 0.262 |
| Chronic lung disease *n (%)* | 11 (1) | 77 (8) | 0.432 |
| Chronic kidney disease *n (%)* | 33 (30) | 216 (22) | 0.059 |
| Congestive heart disease *n (%)* | 22 (20) | 219 (22) | 0.577 |
| Connective tissue disease *n (%)* | 0 (0) | 3 (0) | 0.727 |
| Coronary heart disease *n (%)* | 13 (12) | 156 (16) | 0.608 |
| Dementia *n (%)* | 20 (18) | 185 (19) | 0.863 |
| Diabetes Mellitus *n (%)* | 13 (12) | 135 (14) | 0.572 |
| Liver cirrhosis *n (%)* | 0 (0) | 8 (1) | 0.426 |
| Peripheral arterial disease *n (%)* | 13 (12) | 84 (9) | 0.255 |
| Malignancy *n (%)*  Solid tumor  Leukemia  Lymphoma  Solid tumor with metastases | 10 (9)  0 (0)  1 (1)  1 (1) | 94 (10)  6 (1)  8 (1)  36 (4) | 0.868  0.892  0.918  0.129 |

*Abbreviations:* AIDS, acquired immune deficiency syndrome; n, number; **p-value for Prolonged stay vs. Non-Prolonged stay*

Table 2 – ICU Characteristics of patients with and without prolonged ICU-stay

| *Variables* | *Prolonged stay (≥ 7 days) (n = 110)* | *Non-Prolonged stay (< 7 days) (n = 981)* | *p*-value* |
| --- | --- | --- | --- |
| Laboratory results  Haemoglobin – admission  Leukocytes - admission  Thrombocytes – admission  LDH – admission  Bilirubin – admission (mg/dl)  CRP – admission  Creatinine - admission | 10.4 (9.1 – 11.6)  11.6 (8.5 – 15.9)  206 (165 – 255)  292 (220 – 393)  0.6 (0.4 – 1)  59 (17 – 125)  1.3 (0.8 – 2.0) | 10.3 (9.2 – 11.5)  10.7 (7.9 – 14.1)  207 (153 – 272)  251 (202 – 329)  0.6 (0.4 – 0.9)  27 (8 – 75)  1.1 (0.8 – 1.6) | 0.975  0.057  0.990  0.011  0.679  0.001  0.005 |
| Blood gas analysis  Lactate, mmol/l - admission  pH, level - admission  Base excess – admission  Bicarbonate – admission  paO_2_ – admission  paCO_2_ – admission  pH – nadir  Lactate – peak | 1.3 (0.9 – 2.3)  7.37 (7.32 – 7.44)  - 2 (-6 – 1)  22.6 (20 – 25.4)  101 (79 – 130)  40 (35 – 45)  7.36 (7.27 – 7.49)  2.4 (1.8 – 3.9) | 1.1 (0.8 – 1.7)  7.37 (7.33 – 7.42)  -1 (-4 – 2)  23.6 (21.1 – 25.6)  92 (74 – 132)  41 (37 – 47)  7.36 (7.30 – 7.42)  1.7 (1.1 – 2.6) | 0.002  0.799  0.025  0.070  0.211  0.078  0.227  < 0.001 |

***Data are expressed as n (%) or median (interquartile range);*** ****p-value for Prolonged stay vs. Non-Prolonged stay***
